# A Highly Energetic N‐Rich Zeolite‐Like Metal‐Organic Framework with Excellent Air Stability and Insensitivity

**DOI:** 10.1002/advs.201500150

**Published:** 2015-08-07

**Authors:** Jun‐Sheng Qin, Ji‐Chuan Zhang, Min Zhang, Dong‐Ying Du, Jing Li, Zhong‐Min Su, Yuan‐Yuan Wang, Si‐Ping Pang, Sheng‐Hua Li, Ya‐Qian Lan

**Affiliations:** ^1^ Institute of Functional Material ChemistryDepartment of ChemistryNortheast Normal UniversityChangchun130024P. R. China; ^2^ Jiangsu Key Laboratory of Biofunctional MaterialsSchool of Chemistry and Materials ScienceNanjing Normal UniversityNanjing210023P. R. China; ^3^ School of Materials Science and EngineeringBeijing Institute of TechnologyBeijin100081P. R. China

**Keywords:** air stability, energetic material, insensitivity, N‐rich MOF

## Abstract

**A stable N‐rich aromatic ligand** is employed to prepare energetic zeolite‐like metal‐organic frameworks. **IFMC‐1** shows excellent air stability, and the lowest sensitivity toward impact, friction, and electrostatic discharge and the highest predicted heat of detonation among the reported coordination polymers, and even commercial materials (such as trinitrotoluene (TNT)).

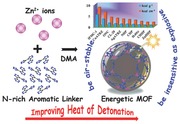

Energetic materials have been used for nearly two centuries not only in military affairs but also in cutting labor costs and expediting laborious processes, which makes a tremendous contribution to the world economy. To harmonize the conflict between energy and safety performance of conventional energetic materials (such as the well‐known TNT (trinitrotoluene)), the research to find more powerful, stable, and reliable energetic materials has been extensively explored. Generally, the excellent energetic materials possess not only high heat of detonation but low sensitivity in consideration of transport, storage, and personal security. The consensus of people at present for enhancing the energy storage is to increase the content of nitrogen element, expecting to prepare N‐rich organic materials.^1^ However, this kind of materials may contain multiple N—N bonds that would increase their sensitivities. Therefore, the search for design synthesis and development of organic explosives with higher nitrogen content and lower sensitivity is one of the most challenging issues in energetic materials.

Metal‐organic frameworks (MOFs) are a new class of porous materials with structural diversity and great potential for various applications in gas storage, fluorescence, separations, sensors, and catalysis.^2^ Recently, a few previous works were involved in the preparation and energetic examination of coordination polymers (CPs, also called MOFs) with 1D–3D structures assembled by coordination bonds between inorganic metal centers and organic linkers.^3^, ^4^, ^5^, ^6^ For instance, Pang group described two energetic 3D MOFs, which exhibit lower sensitivity than the reported 1D and 2D energetic CPs, while possess higher detonation heats than those of the previously reported energetic CPs.^6^ These results suggest that the performance of CPs as high energy materials surpass the related ligand precursors and the concept of designing 3D energetic MOFs has provided a unique architectural platform for developing new‐generation high‐performance explosives. Among numerous MOFs,^7^, ^8^ the zeolite‐like MOFs are good candidates as energetic materials based on the following reasons: (i) the exceptional stability may low the sensitivity of a material, and (ii) most of the zeolite‐like MOFs are composed of N‐containing ligands, which provides a prerequisite for increasing the energy content.^9^ The development of MOF materials as the new‐generation energetic materials is still in its infancy and realization of practically useful MOF materials for their implementation is still challenging. The design synthesis of new promising molecules employed as explosives should not only be insensitive to heat, impact, friction, and electrostatic discharge, as well as resistance to chemical decomposition, but also possesses high detonation heat. Encouraged by these, we intend to employ an N‐rich ligand and expect to obtain energetic zeolite‐like MOFs with high energy and good stability.

As we all know, the stability of N—N bond will be enhanced once it is in an aromatic ring owing to conjugate feature. Therefore, five‐membered N‐containing heterocycles are the main sources of energetic materials because of their high content of nitrogen element and the thermal stability resulting from the aromatic ring system.^10^, ^11^ So far, it is an ideal goal to obtain an organic ligand that possesses high nitrogen content and, meanwhile, avoids the presence of N—N bond of nonaromatic ring. In this context, we prepared an organic ligand with N‐rich aromatic rings, 4,5‐di(1*H*‐tetrazol‐5‐yl)‐2*H*‐1,2,3‐triazole (H_3_dttz; see the Supporting Information).

Continuing the interest in finding new energetic materials with high energy and stability, and eco‐friendly nature, we investigate the synthesis of potential energetic MOF materials with the nitrogen‐rich aromatic linker, H_3_dttz, and Zn(II) cations for the following considerations: (i) as a rigid aromatic ligand, a nitrogen content of H_3_dttz reaches as high as 75.11%, which is higher than other N‐containing heterocyclic rings; (ii) compared with hydrazine and atrz, H_3_dttz has 11 potentially coordinated N atoms per molecule, which is beneficial to the generation of zeolite‐like architectures; and (iii) the diverse coordination modes of Zn(II) ions can form stronger interaction with N atoms, reducing the sensitivity of products. Fortunately, we isolated a 3D zeolite‐like MOF, [Zn(Hdttz)]·DMA (**IFMC‐1**, IFMC = Institute of Functional Material Chemistry; DMA = N,N′‐dimethylacetamide).^12^ Furthermore, the scale‐up experiments were rounded up by successful bulk preparation of **IFMC‐1** with micrometer‐sized shape, which makes it a valuable advance for lab‐scale and industrial production. The impact, friction, and electrostatic discharge sensitivities of **IFMC‐1** were investigated, as well as its heat of detonation. The results suggest that H_3_dttz is a very efficient building block for constructing energetic materials with high energy but low sensitivity.

Colorless micrometer‐sized polyhedral crystals of **IFMC‐1** were isolated by the solvothermal reaction of Zn(II) ions and H_3_dttz in DMA (**Figure** **1**a). The structure of **IFMC‐1** has been reported in our previous work.^12^ The simple descriptions will be given in this section for the convenience of comprehension. In **IFMC‐1**, each Zn(II) center is tetrahedrally coordinated by four nitrogen atoms from three bridging Hdttz^2–^ fragments to complete an infinite 3D neutral framework (Figure 1b). Overall, 63.6% of the N‐donor sites were left unbonded with Zn(II) centers, that is, there are abundant uncoordinated nitrogen atoms from this N‐rich aromatic ligand. Additionally, **IFMC‐1** has remarkable nitrogen content as high as 47.26%, which would be twice more than that of 2D ZnHHP (23.61%). The network of **IFMC‐1** resembles a classical zeolitic SOD topology (Figure 1c,d) and there are six structurally ordered DMA solvent molecules located in each sodalite (SOD) cage (Figure 1e). It is clear to see that **IFMC‐1** formed as a regular micrometer‐sized polyhedron shape under scanning electron microscopy (Figure 1f).

**Figure 1:**
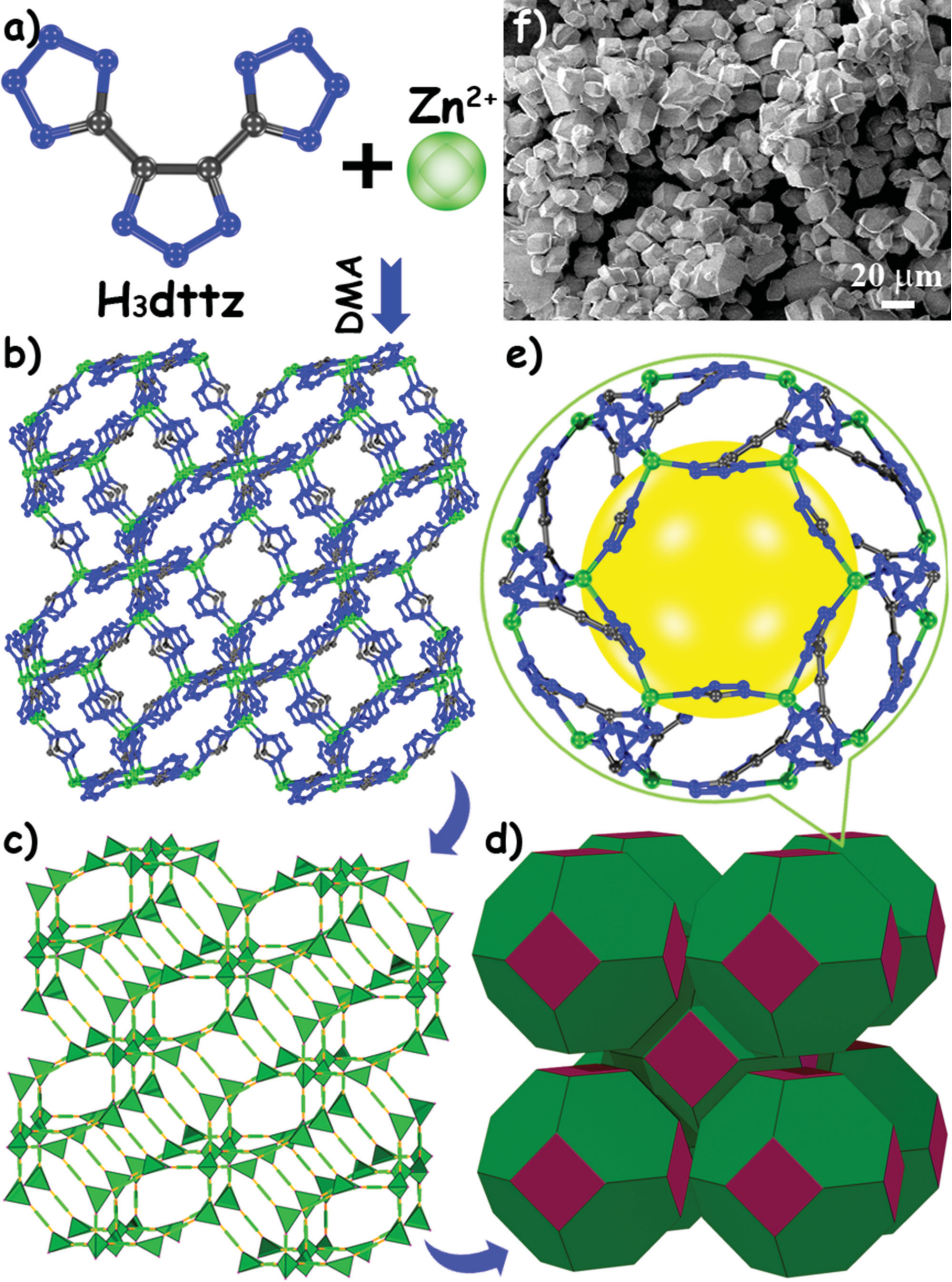
The structure of **IFMC‐1**: a) the precursors, b) the 3D neutral framework, c,d) the SOD topology, e) an SOD cage, and f) the SEM image.

Phase purity of the polyhedral crystals **IFMC‐1** was confirmed by the similarity between the experimental and simulated X‐ray powder diffraction (XRPD) patterns. **IFMC‐1** was stable not only in mother liquor DMA but also in air (**Figure** **2**). Furthermore, it was also found that **IFMC‐1** can be stable in air over 2 years, as confirmed by subsequent XRPD and Fourier transform infrared (FT/IR) measurements (Figure 2 and Figure S1, Supporting Information). The excellent air stability of **IFMC‐1** maybe results from two factors: (i) the coordination bond between metal ion (M) and N atoms from N‐containing aromatic heterocycles is often stronger than that of M—O (carboxylate‐oxygen from organic ligands), and (ii) the structure feature of zeolite‐like MOF. In addition, the thermogravimetric analysis (TGA) and differential scanning calorimetry (DSC) curves of **IFMC‐1** suggest that it exhibits high thermal stability (Figure S2, Supporting Information), which is in agreement with its temperature‐dependent PXRD patterns (Figure S3, Supporting Information). An MOF material with long‐time stability in air is of great importance to be an explosive in practical application.

**Figure 2:**
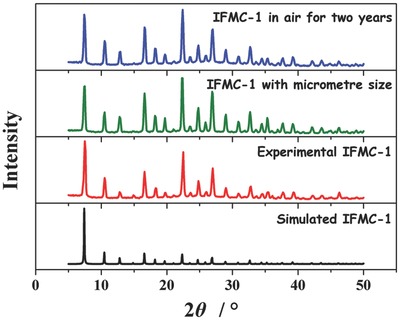
The XRPD patterns of **IFMC‐1**: simulated pattern, freshly prepared sample in DMA, **IFMC‐1** with micrometer‐sized shape, and the as‐synthesized sample in air for more than 2 years.

Furthermore, the structural stability and permanent porosity of micrometer‐sized **IFMC‐1** was also confirmed by the nitrogen adsorption experiments of the guest‐free samples. The activated sample was obtained by exchanging the solvent molecules in the as‐synthesized micrometer‐sized **IFMC‐1** with CH_3_OH and then CH_2_Cl_2_, followed by evacuation under vacuum. The N_2_ sorption isotherm at 77 K exhibits a characteristic type I behavior for microporous materials (Figure S4a, Supporting Information). The absorbance of N_2_ at saturation is ≈244.6 cm^3^ g^–1^ and the Brunauer–Emmett–Teller surface area was 557 m^2^ g^–1^ on the basis of the nitrogen adsorption isotherm. Moreover, pore size distribution was estimated on the N_2_ desorption isotherms (Figure S4b, Supporting Information), which is identical to the result from the single‐crystal X‐ray diffraction study.

The unparalleled stability of **IFMC‐1** encourages us to perform the initial safety testing. The impact, friction, and electrostatic discharge sensitivities of **IFMC‐1** were carried out, as well as H_3_dttz (see the Supporting Information). The data collected are presented in **Table** **1**.^4^, ^5^, ^6^, ^13^ The impact sensitivities of H_3_dttz and **IFMC‐1** are >40 J, classifying them as “insensitive.”^14^ Furthermore, H_3_dttz and **IFMC‐1** are also insensitive to both friction (>360 N) and electrostatic discharge (>40 J), while the electrostatic sensitivities of octogen (HMX), hexogeon (RDX), and hexanitroisowurtzitan (CL‐20) are not more than 0.2 J (Figure S5, Supporting Information). In addition, the 3D zeolitic **IFMC‐1** exhibits significantly lower sensitivity compared with those reported energetic CPs, such as 1D MOFs (CHP, IS = 0.5 J)^4^ and 2D MOFs (CHHP, IS = 0.8 J and ZnHHP, IS = 2.5 J).^5^ (Note: HP = cobalt hydrazine perchlorate, IS = impact sensitivity, CHHP = cobalt hydrazine hydrazinecarboxylate perchlorate, and ZnHHP = zinc hydrazine hydrazinecarboxylate perchlorate). It is probable that the 3D framework of **IFMC‐1** could facilitate the molecules more rigid than that of 1D or 2D structures. Moreover, **IFMC‐1** shows a lower sensitivity than those of the reported 3D MOFs ([Cu(atrz)_3_(NO_3_)_2_]*_n_*, IS = 22.5 J and [Ag(atrz)_1.5_(NO_3_)]*_n_*, IS = 30 J), which maybe result from the rigid aromatic nature of H_3_dttz and the zeolitic framework of **IFMC‐1**. This result once again confirmed that 3D frameworks possess more complicated connection modes, which could further enhance structural reinforcement, hence improve the stabilities and energetic properties.^6^

**Table 1:** Physicochemical properties of H_3_dttz and **IFMC‐1**

Entry	*T*_d_ a)	*D*_c_ b)	N%c)	ISd)	FSe)	EDSf)
H_3_dttz	300	1.75	75.11	>40	>360	>40
**IFMC‐1**	392	1.47	47.26	>40	>360	>44
CHP^4^	194	1.95	14.71	0.5	–	–
CHHP^5^	231	2.00	28.25	0.8	–	–
ZnHHP^5^	293	2.12	23.61	2.5	–	–
atrz^6^	313	1.62	68.27	14	180	>10.12
[Cu(atrz)_3_(NO_3_)_2_]*_n_* 6	243	1.68	53.35	22.5	112	24.75
[Ag(atrz)_1.5_(NO_3_)]*_n_* 6	257	2.16	43.76	30	84	>24.75
TNT^13^	295	1.65	18.50	15	353	–
HMX^13^	–	1.91	37.84	7.4	112	0.20
TATB^13^	318	1.94	32.55	50	–	–
RDX^13^	–	1.81	37.84	7.5	120	0.20
CL‐20^13^	–	2.04	38.3	4	48	0.13

^a)^Decomposition temperature (DSC, °C);

^b)^Density from X‐ray diffraction analysis (g cm^−3^);

^c)^Nitrogen content;

^d)^Impact sensitivity (J);

^e)^Friction sensitivity (N);

^f)^Electrostatic sensitivity (J).

Working as an energetic material, the heat of detonation (*∆H*_det_) is a critical parameter for consideration of its performance. In order to estimate the detonation heat of this new explosive and compare the ∆*H*_det_ value with those of documented 1D, 2D, and 3D energetic MOF materials, we chose the same methodology recently employed for nickel hydrazine perchlorate (NHP) and CHP.^4^ Furthermore, we also employ density functional theory to compute the energy of detonation (∆*E*_det_), from which the ∆*H*_det_ value is estimated by using a linear correlation developed from known ∆*H*_det_ data for commonly used high explosives.

For **IFMC‐1**, the formation of metal oxide was assumed to be governed by the deficiency of oxygen, and nitrogen, carbon, and ammonia were assumed to be the final products of decomposition of the organic moiety. The complete detonation reaction is described by Equation (1) as follows (1)
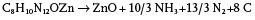



The predicted heat of detonation of **IFMC‐1** is 5.62 kcal g^−1^ (8.26 kcal cm^−3^), which is five times more than that of H_3_dttz (1.06 kcal g^−1^; **Figure** **3**). In addition, the heat of detonation of **IFMC‐1** is much higher than those of the most powerful organic explosives known (TNT, 1.0 kcal g^−1^, CL‐20, about 1.5 kcal g^−1^, and octanitrocubane (ONC), about 1.8 kcal g^−1^).^15^ The detonation heat of **IFMC‐1** is superior to all of the reported energetic MOF materials, for instance, CHP (≈1.25 kcal g^−1^), NHP (≈1.37 kcal g^−1^), CHHP (≈0.75 kcal g^−1^), [Cu(atrz)_3_(NO_3_)_2_]*_n_* (3.62 kcal g^−1^), and [Ag(atrz)_1.5_(NO_3_)]*_n_* (1.38 kcal g^−1^). To our knowledge, **IFMC‐1** possesses the highest heat of detonation among all of the previously reported metal‐based energetic MOFs (Figure 3b). It is also worth pointing out that advancements in energetic materials were driven by a need to find more powerful, stable, and reliable materials for diverse commercial applications. **IFMC‐1** retains good stability in the air for a long time and can be prepared with high yield, making it a competitive highly energetic material.

**Figure 3:**
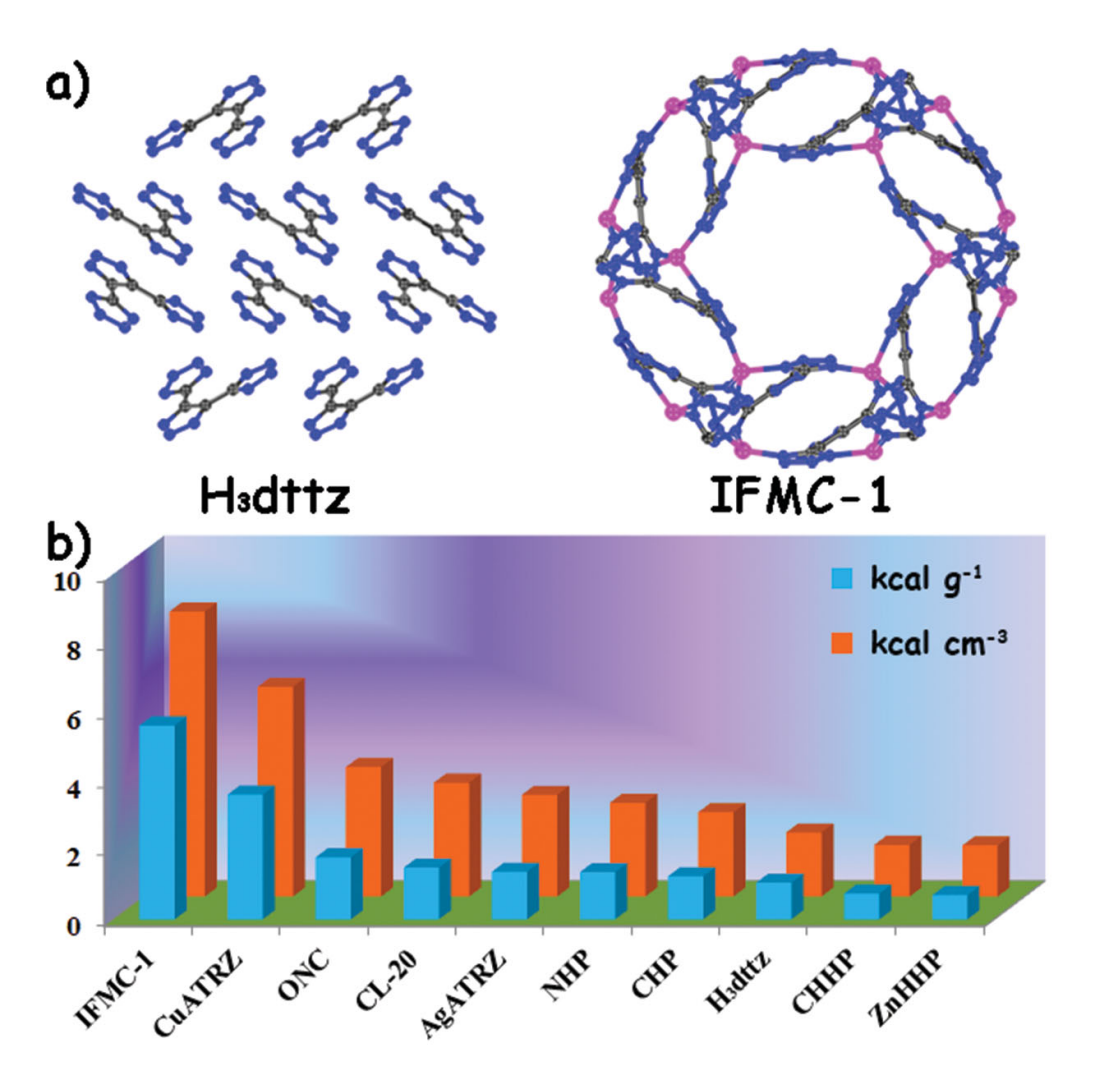
a) The comparison of H_3_dttz and **IFMC‐1** and b) bar diagram representation of the Δ*H*_det_ values for the common explosive materials including octanitrocubane (ONC), hexanitrohexaazaisowurtzitane (CL‐20), and previously reported values for energetic MOFs along with the predicted Δ*H*_det_ values for **IFMC‐1** and H_3_dttz are also shown.

**IFMC‐1** shows the lowest sensitivity toward impact, friction, and electrostatic discharge among the reported 1D and 2D energetic MOFs, while its calculated value of detonation heat is higher than all of the previously reported energetic MOFs, and even commercial materials (such as TNT, CL‐20, and ONC). Crystallographic study reveals that **IFMC‐1** possesses a 3D porous zeolite‐like MOF structure with SOD topology, which perhaps results in the high air stability of **IFMC‐1**. As in the documented literature, the zeolitic metal‐imidazolate frameworks, as the prominent subfamily of zeolite‐like MOFs, are noteworthy for their exceptional stability and have potential application prospects in diverse areas. Compared with H_3_dttz and the reported 1D and 2D CPs, energetic 3D MOF, **IFMC‐1** possesses more complicated connection modes and enhanced structural reinforcement. The relatively high heat of detonation perhaps results from the nitrogen‐rich heterocyclic aromatic ligand and the fascinating zeolite‐like motif,^16^ the 3D cage of **IFMC‐1** has high strain energies locked in the molecules, and it is released as an additional energy on detonation. In addition, the stable 3D MOF with channel structure owns larger specific surface area, leading to a high heat of donation. To some extent, this is another advantage compared with 1D and 2D energetic MOF materials.

In conclusion, a 3D zeolite‐like N‐rich energetic MOF, **IFMC‐1**, with SOD topology was achieved by the self‐assembly of a stable N‐rich organic ligand (H_3_dttz) and nontoxic metal ion. Furthermore, **IFMC‐1** can be stable in air for more than 2 years and exhibits extreme insensitivity to the impact, friction, and electrostatic discharge. As an energetic explosive, the predicted heat of detonation of 3D zeolitic MOF, **IFMC‐1**, was five times more than that of its precursor, H_3_dttz. This result maybe result from **IFMC‐1** possessing more complicated connection modes and enhanced structural reinforcement than H_3_dttz. Furthermore, the calculated value of detonation heat of **IFMC‐1** is higher than other reported metal‐based energetic compounds, and even three to five times higher than those of CL‐20, ONC, and TNT. It is an efficient way to employ an N‐rich aromatic organic ligand to prepare zeolite‐like MOFs, which enhances the stability and increases the heat of detonation of the materials. Energetic **IFMC‐1** is a promising candidate for replacing heavy‐metal‐based explosives, featuring N‐rich zeolite‐like MOF in the absence of sensitive anions. This work not only illustrates a successful case of preparing potential MOF‐based energetic material but also provides a new insight into the design and synthesis of a new generation of green 3D metal‐organic explosives. We are convinced that this newly emerging field is bubbling with opportunities and that further work will be made in our lab.

## Experimental Section

*Preparation of Micrometer‐Sized **IFMC‐1***: H_3_dttz (15.37 g, 75 mmol) was dissolved in DMA (80 mL) with ultrasonic dispersion, and then Zn(NO_3_)_2_·6H_2_O (44.55 g, 150 mmol) was added to the resulted solution. The mixture was heated at 95 °C for 3 d and then cooled to room temperature. Colorless polyhedral microcrystals were collected, washed with anhydrous DMA for three times, and then dried in air (yield 86%, based on Zn(NO_3_)_2_·6H_2_O). IR (KBr, cm^–1^, Figure S1, Supporting Information): 3439 (m), 2938 (m), 1929 (m), 1606 (s), 1436 (s), 1261 (m), 1175 (m), 1066 (m), 1030 (s), 1004 (m), 971 (m), 942 (m), 752 (m), 683 (w), 608 (m), 506 (s).

## Supporting information

As a service to our authors and readers, this journal provides supporting information supplied by the authors. Such materials are peer reviewed and may be re‐organized for online delivery, but are not copy‐edited or typeset. Technical support issues arising from supporting information (other than missing files) should be addressed to the authors.

Supplementary
