# Supplementary material for: A Highly Energetic N‐Rich Zeolite‐Like Metal‐Organic Framework with Excellent Air Stability and Insensitivity
Source: Adv Sci (Weinh). 2015 Aug 7;2(12):1500150. doi: 10.1002/advs.201500150 (PMC5115308; doi:10.1002/advs.201500150)
Supplement: Supplementary file 1 — Supplementary [file ADVS-2-0a-s001.pdf]

Copyright WILEY-VCH Verlag GmbH & Co. KGaA, 69469 Weinheim, Germany, 2015.

ADVANCED SCIENCE
Open Access

Supporting Information

for *Adv. Sci.*, DOI: 10.1002/advs. 201500150

A Highly Energetic N-Rich Zeolite-Like Metal-Organic Framework with Excellent Air Stability and Insensitivity

Jun-Sheng Qin, Ji-Chuan Zhang, Min Zhang, Dong-Ying Du, Jing Li, Zhong-Min Su, Yuan-Yuan Wang, Si-Ping Pang, Sheng-Hua Li,* and Ya-Qian Lan**

Copyright WILEY-VCH Verlag GmbH & Co. KGaA, 69469 Weinheim, Germany, 2013.

Supporting Information

A Highly Energetic N-rich Zeolite-like Metal-Organic Framework with Excellent Air-Stability and Insensitivity

Jun-Sheng Qin, Ji-Chuan Zhang, Min Zhang, Dong-Ying Du, Jing Li, Zhong-Min Su, Yuan-Yuan Wang, Si-Ping Pang, Sheng-Hua Li,* and Ya-Qian Lan**

Institute of Functional Material Chemistry, Department of Chemistry, Northeast Normal University, Changchun 130024, P. R. China
School of Chemistry and Materials Science, Nanjing Normal University, Nanjing 210046, P. R. China
School of Materials Science & Engineering, Beijing Institute of Technology, Haidian District, Beijing 100081, P. R. China

S1. Materials and Measurements

The ligand H_3dttz was synthesized according to modified procedure of the reported literature.[1] All other chemicals were obtained from commercial sources, and were used without further purification. Single crystal X-ray diffraction data were recorded on a Bruker APEXII CCD diffractometer with graphite-monochromated Mo Kα radiation (λ = 0.71069 Å) at 293 K. IR spectrum was performed in the range 4000–400 cm^{-1} using KBr pellets on an Alpha Centaurt FT/IR spectrophotometer. The X-ray powder diffraction (XRPD) data were collected on a Bruker D8 Advance diffractometer with Cu-Kα (λ = 1.5418 Å) ranging from 5 to 50° at room temperature. Thermogravimetric analysis (TGA) of the samples was recorded using a Perkin-Elmer TG-7 analyzer heated from room temperature to 800 °C under nitrogen at the heating rate of 5 °C·min^{-1}. Field-emission scanning electron microscopy (FE SEM) images were obtained with a XL30 ESEM FEG microscope. The N_2 sorption measurements were performed on automatic volumetric adsorption equipment (Quantachrome Autosorb-iQ).

S2. The Synthesis of H_3dttz

A mixture of 1*H*-1,2,3-triazole-4,5-dicarbonitrile (11.9 g, 100 mmol), NaN_3 (26.0 g, 400 mmol), and triethylamine hydrochloride (68.8 g, 500 mmol) in 250 mL of toluene and 50 mL of methanol was heated at reflux in a 500-mL round-bottom flask for 3 days. Upon cooling to room temperature, 200 mL of an aqueous solution of NaOH (2.5 M) was added, and the mixture was stirred for 30 min. The aqueous layer was treated with ca. 140 mL of HCl (3 M) until no further white precipitate formed. The precipitate was then collected by filtration, dried in the air, and dissolved in aqueous NaOH (1 M). The resulting clear, colorless solution was titrated with diluted HCl (1 M) until the pH of the solution was 4-5. The ensuing white precipitate was washed with successive aliquots of distilled water (500 mL), methanol (300 mL), and acetone (50 mL) to afford 16.8 g (82%) of product. ^{13}C NMR (126 MHz, d_6-DMSO) δ 131.47, 150.76. IR (KBr, cm^{-1}, Figure S6): 3505 (s), 3024 (m), 1850 (w), 1655 (m), 1587 (s), 1447 (s), 1232 (w), 1249 (w), 1211 (m), 1128 (w), 1089 (w), 995 (s), 962 (m), 867 (m), 748 (w), 694 (m), 586 (s), 541 (m).

S3. Sensitivity

Impact Sensitivity: The impact sensitivity was tested on a type 12 tooling according to "up and down" method (Bruceton method). A 2.5 kg weight was dropped from a set height onto a 20 mg sample placed on 150 grit garnet sandpaper. Each subsequent test was made at the next lower height if explosion occurred and at the next higher height if no explosion happened. 50 drops were made from different heights, and an explosion or non-explosion was recorded to determine the results. RDX was considered as a reference compound, the impact sensitivity of RDX is 7.4 J.

Friction Sensitivity: 20 mg sample was placed on a Козпов apparatus. 25 tests were done. An explosion or non-explosion was recorded. RDX was considered as a reference compound, and the friction sensitivity of RDX is 76%. The friction sensitivities of 1 and 2 are 0 %. Test conditions: 20 °C (temperature); 28% (relative humidity); 90° (swing angle); 474.6 MPa (test pressure).

Electrostatic Sensitivity: When electrostatic sensitivity was considered, 25 mg sample was placed on a JGY-50(Ⅲ) Electrostatic test apparatus, while the high voltage was supplied by an EST806F Electrostatic Power Generator. The voltage was increased gradually from 1 kV to 15 kV. No explosion occurred. Therefore, 25 trials were done when the voltage was 15 kV. 1 explodes at the voltage of 15 kV, while 2 shows no sensitivity to the voltage of 15 kV, which is the limit range of the apparatus. Test conditions: 25 °C (temperature); 34% (relative humidity); Capacitance: 0.22 μF.

S4. Heat of Detonation

Density functional theory (DFT) was used to compute the energy of detonation (ΔE_{det}), from which ΔE_{det} is estimated by using a linear correlation equations (ΔE_{det} = 1.127 ΔE_{det} + 0.046, r = 0.968). As in the reported literature,[2]the DFT calculations for energetic MOF (**IFMC-1**) were performed with the code DMOl3[3]under 3D periodic boundary conditions employing the Monkhorst-Pack multiple K-point sampling of the Brillouin zone[4]and the Perdew-Becke-Ezerhoff (PBE) exchange-correlation function.[5]The complete detonation reactions are described by Equation (1).

$$C_8H_{10}N_{12}OZn \rightarrow ZnO + 10/3\ NH_3 + 13/3\ N_2 + 8\ C \qquad (1)$$

Table S1. Calculated parameters used in the detonation reactions

$C_8H_{10}N_{12}OZn$(Ha)	ZnO(Ha)	NH_3(Ha)	N_2(Ha)	C(Ha)
–1269.5552	–302.2495	–56.5017	–109.4479	–37.738

S5. Heat of Formation

Isodesmic reaction, in which numbers of electron pairs and chemical bond types are conserved, has been employed very successfully to give heat of formation more accurate than semi-empirical calculation.[6] Based on the optimized structures, the total energy (E_0) and thermodynamic parameters, including zero point energy (ZPE) and thermal correction to enthalpy (H_T), were obtained at the B3LYP/6-311++g(d, p) level.

For the isodesmic reaction (Scheme S1), heat of reaction (ΔH_{298K}) can be calculated from the following equation (2):

$$\Delta H_{298\text{K}} = \Delta H_{\text{f,P}} - \Delta H_{\text{f,R}} \qquad (2)$$

where $\Delta H_{f,R}$ and $\Delta H_{f,P}$ are the heats of formation for reactants and products at 298.15 K, respectively. Meanwhile, ΔH_{298K} can also be calculated using the following equation (3):

$$\Delta H_{298K} = \Delta E_{298K} + \Delta(PV) = \Delta E_0 + \Delta ZPE + \Delta H_T + \Delta(nRT) \quad (3)$$

Where ΔE_0 is the change in total energy between the products and the reactants at 0 K; ΔZPE is the difference between the zero-point energies of the products and the reactants; ΔH_T is thermal correction from 0 K to 298.15 K. Since there is no change in number of total molecules, Δ(PV) = Δ(nRT) = 0. Therefore, the heat of formation can be figured out according to ΔH_{298K} and heats of formation of other reactants and products. Fortunately, these data can be acquired from the literature and handbook facilely.

Scheme S1. Isodesmic reaction

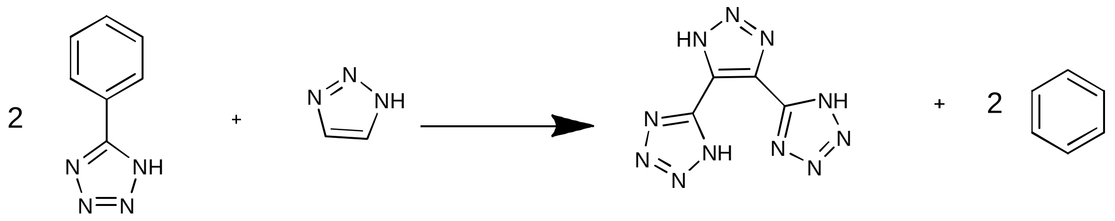

Table S2. Calculated total energy (E_0), zero-point energy (ZPE), thermal correction (H_T), and enthalpy of formation (HOF) of azole-based compounds and reference compound

Compounds	E_0 (a.u.)	ZPE (kJ/mol)	H_T (kJ/mol)	HOF (kJ/mol)
5-phenyl-1*H*-tetrazole	–489.701167	360.05	25.16	413
1*H*-1,2,3-Triazole	–242.292758	154.25	11.96	272[7]
Benzene[8]	–232.248647	264.51	14.00	82.9

S6. Detonation Performances

Detonation performance of the related energetic MOF (**IFMC-1**) here was evaluated by the empirical Kamlet formula,[9] as

$$D = 1.01\ \Phi^{1/2}\ (1+1.30\rho)$$
$$P = 1.558\ \Phi\rho^2$$
$$\Phi = 31.68\ N(MQ)^{1/2}$$

where D represents detonation velocity (km·s^{-1}) and P is detonation pressure (GPa), ρ is the density of explosive (g·cm^{-3}). Φ, N, M and Q are characteristic parameters of an explosive. N is the moles of detonation gases per gram of explosive, M is the average molecular weight of these gases and Q is the heat of detonation (kcal·g^{-1}). The complete detonation reactions are described by Equation (1). The formation of metal oxides as solid was assumed to be governed by the deficiency of oxygen.

Table S3. The comparison of detonation properties of some energetic MOFs with **IFMC-1**

explosive	ρ ($g \cdot cm^{-3}$)	N ($mol \cdot g^{-1}$)	M ($g \cdot mol^{-1}$)	Q ($kcal \cdot g^{-1}$)	D ($km \cdot s^{-1}$)	P (GPa)
H_3dttz	1.75[a]	0.0293	26.167	1.063	8.40	26.63
**IFMC-1**	1.468	0.0216	23.197	5.620	8.21	26.23
$[Cu(atrz)_3(NO_3)_2]_n$	1.68	0.027	24.873	3.618	9.160	35.68
$[Ag(atrz)_{1.5}(NO_3)]_n$	2.16	0.022	24.870	1.381	7.773	29.70
CHP	1.948	0.033	21.073	~1.25	8.225[b]	31.73[b]
NHP	1.983	0.038	21.073	~1.37	9.184[b]	39.69[b]
CHHP	2.00	0.028	14.069	~0.75	6.205	17.96
ZnHHP	2.117	0.026	24.000	~0.7	7.016	23.58
TNT[10]	1.654	—	—	1.0	6.881	19.53
HMX[10]	1.910	—	—	—	9.320	39.63
TATB[10]	1.937	—	—	—	8.114	31.15

[a] Density measured by gas pycnometer (25 °C). [b] The detonation velocity and detonation pressure were evaluated by the empirical Kamlet formula.

S7. The Supporting Figures

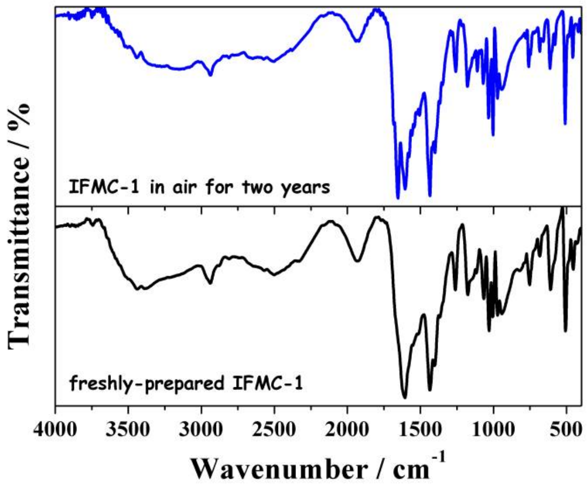

Figure S1. The IR spectra of **IFMC-1** measured in KBr pellets from 4000 cm^{-1} to 400 cm^{-1}.

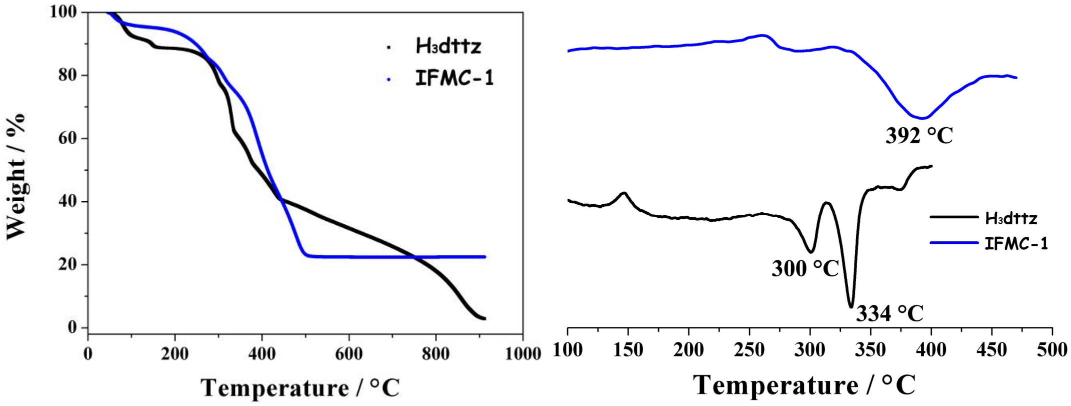

Figure S2. The TGA (*left*) and DSC (*right*) curves for **IFMC-1** and H_3dttz recorded under N_2 atmosphere from room temperature to 900 °C at the heating rate of 5 °C·min^{-1}.

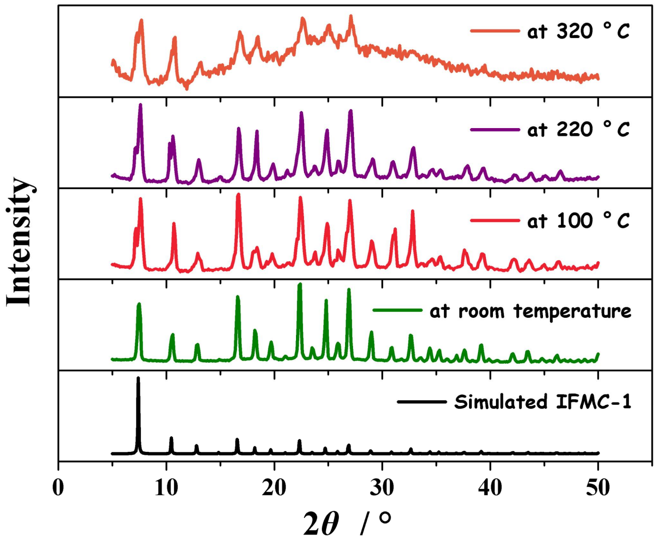

Figure S3. The temperature-dependent PXRD patterns of **IFMC-1**.

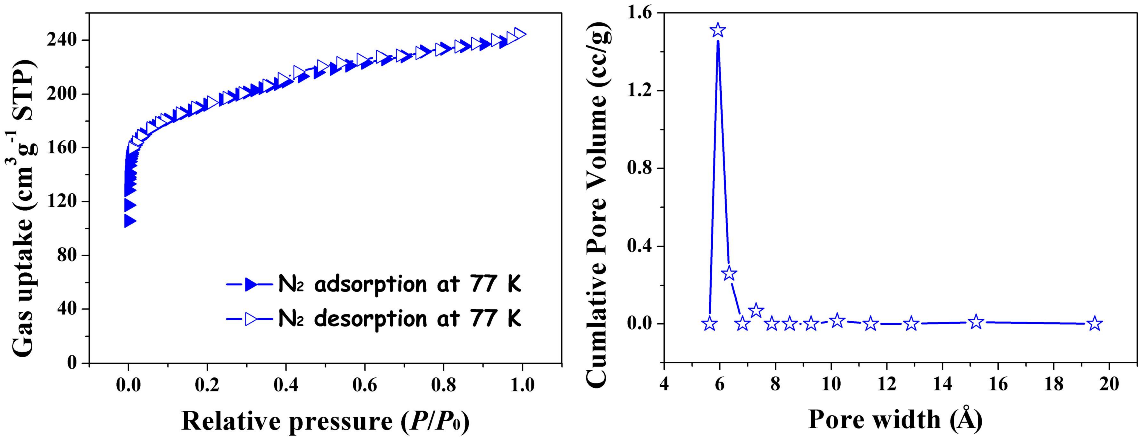

Figure S4. The nitrogen sorption isotherms for μm-sized **IFMC-1a** recorded at 77 K (*left*) and the pore size distribution (*right*), respectively.

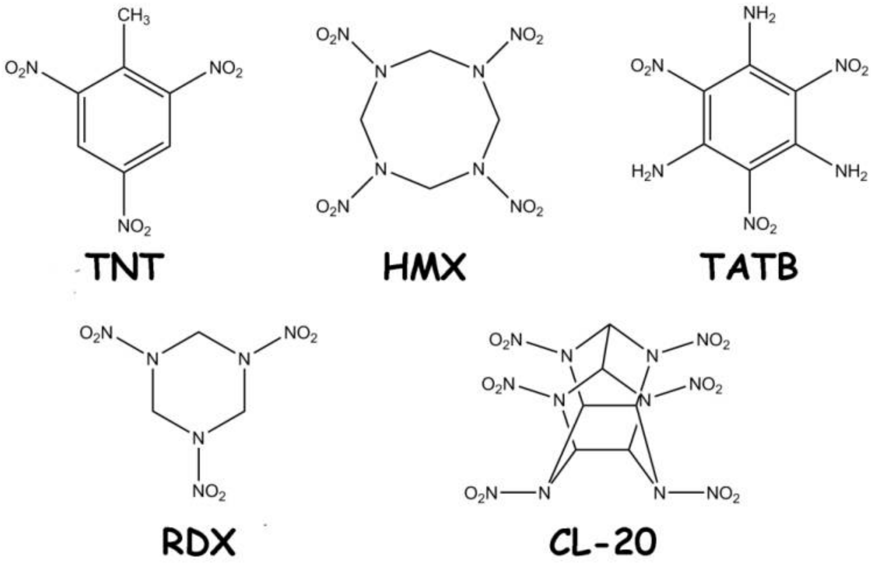

Figure S5. The structures of five common explosive materials: trinitrotoluene (TNT), cyclotetramethylenetetranitramine (HMX), triaminotrinitrobenzene (TATB), cyclotrimethylenetrinitramine (RDX), and hexanitrohexaazaisowurtzitane (CL-20).

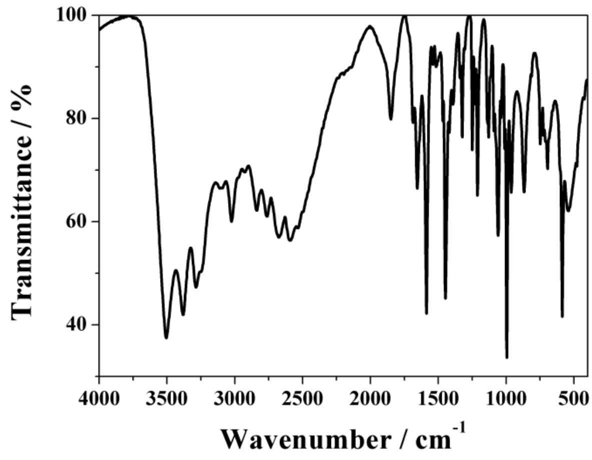

Figure S6. The IR spectrum of H_3dttz measured in KBr pellets from 4000 cm^{-1} to 400 cm^{-1}.

References

[1] M. Dincă, A. Dailly, Y. Liu, C. M. Brown, D. A. Neumann, J. R. Long, *J. Am. Chem. Soc.* **2006**, *128*, 16876.

[2] O. S. Bushuyev, P. Brown, A. Maiti, R. H. Gee, G. R. Peterson, B. L. Weeks, L. J. Hope-Weeks, *J. Am. Chem. Soc.* **2012**, *134*, 1422.

[3] B. J. Delley, *Chem. Phys.* **1990**, *92*, 508.

[4] H. J. Monkhorst, J. D. Pack, *Phys. Rev. B* **1976**, *13*, 5188.

[5] J. P. Perdew, K. Burke, M. Ernzerhof, *Phys. Rev. Lett.* **1996**, *77*, 3865.

[6] a) M. J. Kamlet, S. J. Jacobs, *J. Chem. Phys.* **1968**, *48*, 23; b) R. C. Bingham, M. J. S. Dewar, D. H. Lo, *J. Am. Chem. Soc.* **1975**, *97*, 1285.

[7] A. Padwa, B. M. Trost, I. Fleming, *Comprehensive Organic Synthesis*, 4, Pergamon Press, Oxford, **1991**.

[8] W. J. Hehre, L. Radom, P. V. R. Schleyer, J. A. Pople, *Ab initio molecular orbital theory*, Wiley: New York, NY, USA, **1986**. http://onlinelibrary.wiley.com/doi/10.1002/jcc.540070314/pdf.

[9] M. V. Roux, M. Temprado, J. S. Chickos, Y. Nagano, *J. Phys. Chem. Ref. Data* **2008**, *37*, 1855.

[10] U. Teipel, *Energetic Materials*, Wiley-VCH, Weinheim, **2005**.
